# A novel mouse line with epididymal initial segment-specific expression of Cre recombinase driven by the endogenous *Lcn9* promoter

**DOI:** 10.1371/journal.pone.0254802

**Published:** 2021-07-26

**Authors:** Qian-qian Gong, Xiao Wang, Zhi-lin Dou, Ke-yi Zhang, Xiang-guo Liu, Jian-gang Gao, Xiao-yang Sun

**Affiliations:** Shandong Provincial Key Laboratory of Animal Cell and Developmental Biology, School of Life Sciences, Shandong University, Qingdao, China; University of Hyderabad, INDIA

## Abstract

Spermatozoa released from testes undergo a maturation process and acquire the capacity to fertilize ova through epididymal transit. The epididymis is divided into four regions, each with unique morphology, gene profile, luminal microenvironment and distinct function. To study the functions of relevant genes in the epididymal initial segment (IS), a novel IS-specific mouse model, Lcn9-Cre knock-in (KI) mouse line was generated via CRISPR/Cas9 technology. The TAG stop codon was replaced by a 2A-NLS-Cre cassette, resulting in the co-expression of Lcn9 and Cre recombinase. IS-specific Cre expression was first observed from postnatal day 17. Using the Rosa26^tdTomato^ reporter mice, the Cre-mediated DNA recombination was detected exclusively in principal cells. The epididymal IS-specific Cre activity *in vivo* was further confirmed using Lcn9-Cre mice crossed with a mouse strain carrying *Tsc1* floxed alleles (*Tsc1*^flox/+^). Cre expression did not affect either normal development or male fecundity. Different from any epididymis-specific Cre mice reported previously, the novel Lcn9-Cre mouse line can be used to introduce entire IS-specific conditional gene editing for gene functional study.

## Introduction

Infertility affects about 15% of the global population, and approximately half of the cases are attributed to a “male factor” [1, 2]. In male infertility, sperm dysfunction is considered as the most principal cause [3]. Impaired epididymal spermatozoa maturation is an important cause of sperm dysfunction [4]. Following testicular spermatogenesis, immature and nonfunctional spermatozoa pass through the efferent ducts into the epididymis and undergo a complex maturation process through epididymal transit [3, 5].

The epididymis is a conserved part of the male reproductive tract in all vertebrate species that practice internal fertilization [6–8]. The studies carried out in laboratory animal models are meaningful to our understanding of the structure, function, physiology and pathophysiology of human epididymis [9, 10]. In rodents, the epididymis is composed of a highly convoluted tubule, further divided into four distinct regions from proximal to distal: the initial segment (IS), caput, corpus and cauda [11–14], in which the major specialized epithelial cells, principal cells, clear cells, narrow cells and basal cells, distribute in a region-specific manner to establish region-specific luminal environments and distinctive functions [3, 15]. Furthermore, genes are highly ordered and compartmentalized in discrete epididymal segments [16–18]. With specific cell composition, unique morphology, and gene profile, each region carries out a distinct luminal fluid environment and distinctive maturational events. Until now, the molecular and biochemical events essential for epididymal sperm maturation have remained largely unknown.

Genetically engineered mouse models have been widely used to study the functions of epididymis-expressed genes in sperm maturation. For many ubiquitous genes, mutations by conventional gene targeting methods may increase the risk of embryonic mortality and premature death. Thus, conditional ablation of genes in particular epididymal regions with the Cre/*loxP* system is an ideal approach to annotating gene functions, and construction of different epididymal region-specific Cre mouse models is important. The emergence of clustered regularly interspaced short palindromic repeats (CRISPR)/Cas9 technique with higher efficiency and reduced cost, has opened a new era in the generation of Cre mouse lines [19].

Several epididymis-specific Cre mouse lines have been established. Two caput-specific Cre mouse lines by the *Lcn5* promoter were generated through a transgenic strategy, one of which is tamoxifen-inducible [20, 21]. In the other mouse lines (Defb41-Cre [22, 23], Rnase10-Cre [24] and Crisp4-Cre [25]), the NLS-Cre cassettes inserted into the endogenous gene loci were reported to mediate gene editing in the IS, but few could accomplish the editing in the entire IS region. Thus, even though the IS is considered important for male fertility, the understanding of gene functions related to IS in epididymal sperm maturation remains limited due to the lack of effective models.

The murine epididymal secretory protein LCN9 belongs to the evolutionarily conserved lipocalin (Lcn) family [26, 27]. *Lcn9* expression is dependent on the presence of circulating testicular factors in the epididymal lumen, and detectable as early as postnatal 3 weeks [26], and it exhibits a conserved IS-specific distribution pattern in mice and rat [26]. Disruption of endogenous *Lcn9* in male mice showed normal spermatogenesis, sperm maturation and fertility [28]. Here, a novel KI mouse model was established through CRISPR/Cas9 technology, in which the endogenous *Lcn9* promoter directs the IS-specific expression of Cre recombinase. It is a promising tool for use in generation of conditional knock-out mice and for functional studies of relevant genes in sperm maturation, specifically in the IS.

## Materials and methods

### Animals

All animal experiments were approved by the Ethics Committee for Animal Research of School of Life Sciences of Shandong University (permit number: SYDWLL-2018-18) and carried out accordingly. All surgery was performed under sodium pentobarbital anesthesia, and all efforts were made to minimize suffering. C57BL/6J mice were housed in individually ventilated cage systems under a strictly controlled environment (22–24°C; 12h light/dark cycle), with ad libitum access to standard chow. B6.Cg-Gt(ROSA)26Sor^tm9(CAG-tdTomato)Hze/J^ (Rosa26^tdTomato^) reporter mice [29], obtained from The Jackson Laboratory, were crossed with Lcn9-Cre mice to generate Lcn9-Cre; Rosa26^tdTomato^ males. *Tsc1*^flox/+^ mice [30] were imported from The Jackson Laboratory, and mated with Lcn9-Cre mice to establish Lcn9-Cre; *Tsc1*^flox/+^ mice. Genomic DNA was isolated from tails and genotype was identified through polymerase chain reaction (PCR) followed by electrophoresis with a DNA ladder (250 bp-I DNA ladder; MD Bio). Primers for genotyping are listed in S1 Table.

### Construction of sgRNA and donor vector

A pair of oligonucleotides against *Lcn9* sgRNA (5’-TTGCTTTTTATAGACCATAGAGG-3’) were annealed and cloned into the *pX330* plasmid (42230; Addgene) through BbsI site. Cas9 mRNA and sgRNA were synthesized with a mMESSAGE mMACHINE T7 transcription kit (AM1344; Life Technologies) and MEGAshortscript T7 transcription kit (AM1354; Life Technologies), respectively.

For donor vector construction, 2A self-cleaving peptides-nuclear localization signal -Cre (2A-NLS-Cre) sequence, 1.0 kb 5’-homology arm, the 970 bp 3’-homology arm, and other DNA fragments were pooled together and assembled by a “T5 exonuclease DNA assembly” method [31]. The donor vector was digested by KpnI and EcoRI. Linearized DNA fragments were recovered and purified.

### Generation of Lcn9-Cre KI mice

Cas9 mRNA, sgRNA, and linearized donor vectors were co-injected into fertilized zygotes to generate targeted Lcn9-Cre KI offspring. F0 founders were identified by PCR, which were bred to wild-type (WT) mice to test germline transmission and F1 animal generation. The insertion was amplified and further confirmed by sequencing.

### Southern blotting

Southern blotting was performed as described previously [32]. Genomic DNA was extracted from tails and digested by SacI. DNA fragments were electrophoresed on a 0.8% agarose gel and transferred to a nylon membrane. A probe targeting the Cre element was synthesized with the PCR DIG Probe Synthesis Kit (Roche). The membrane was hybridized with the Dig-labeling probe and detected with a DIG DNA Labeling and Detection Kit (Roche).

### RT-PCR

Total RNA was extracted with TRIzol reagent (15596018; Invitrogen), genomic DNA was removed, and the complementary DNA (cDNA) was synthesized with the PrimeScript™ RT reagent Mix (RR0047A; TAKARA). The reverse-transcribed product was diluted and utilized to amplify the genes of interest (GAPDH served as the internal reference; primers listed in S1 Table). Tissues obtained from more than 3 different animals in each group were analyzed.

### Western blotting

Total protein lysates were isolated, separated with SDS-PAGE and transferred to a 0.45 μm PVDF membrane (Millipore). Blots were probed with anti-GAPDH (60004-1-Ig; rabbit; Proteintech; 1: 20,000) [33], anti-TSC1 (6935; rabbit; CST; 1:1000) [34], and anti-Cre recombinase (15036; rabbit; CST; 1:1000) [35] antibodies, developed with an HRP conjugated goat anti-rabbit secondary antibody (BA1054; Boster-bio; 1:10000) and detected with an electrochemiluminescence (ECL) detection kit (32106; Thermo). More than 3 mice were included in each group. The bands were quantified using the ImageJ software (NIH).

### Immunofluorescence imaging

To investigate the spatial distribution of active Cre recombinase, tdTomato signals in various tissues from 5-month-old Lcn9-Cre; Rosa26^tdTomato^ mice were examined with a fluorescence stereomicroscope (M165; Leica). To determine changes over time, tdTomato signals in Lcn9-Cre; Rosa26^tdTomato^ mice of different ages were detected.

To visualize the precise distribution of active Cre recombinase and track the cell types with positive signals, epididymal samples isolated from the heterozygous Lcn9-Cre; Rosa26^tdTomato^ males were embedded in Tissue-Tek OCT (4583; Sakura). Six-μm thick frozen sections were washed with phosphate-buffered saline (PBS), blocked with 10% goat serum and incubated with primary antibodies overnight at 4°C. Immunofluorescence signals were screened after FITC-conjugated secondary antibody incubation and counterstaining with DAPI. Primary antibodies included cytokeratin 5 (Krt5) (ab52635; rabbit; Abcam; 1:200) [36] for basal cells, α-SMA (BM0002; mouse; Boster-bio; 1:200) for smooth muscle cells, and vacuolar H^+^-ATPase (V-ATPase) (ab200839; rabbit; Abcam; 1:200) [37] for narrow cells and clear cells. FITC-conjugated goat anti-mouse IgG (ZF-0312; ZSGB-bio; 1:100) and goat anti-rabbit IgG (ZF-0311; ZSGB-bio; 1:100) were used as secondary antibodies. Fluorescent images were captured with a confocal microscope (LSM900; ZEISS).

### Histological analysis and imaging

Testes and epididymides, collected from six-month-old Cre KI males and WT controls, were visualized and imaged with a stereomicroscope, fixed in the Bouin’s fluid and embedded in paraffin. Five-μm sections were stained with hematoxylin and eosin (H&E) according to the standard protocols. Sperm were collected from cauda, spread on slides, fixed and then stained with H&E solution.

### Mating tests

To study male fertility, two-month-old homozygous Lcn9-Cre KI males and WT male littermates were mated with C57BL/6J females. One male was caged with 2 females in each cage for 4 months. The number of offspring and litters was recorded. Four homozygotes and four WT controls were used in this experiment.

### Statistical analysis

Almost all experiments in this study were conducted at least three times using different batches of mice. Representative images are shown in the corresponding figures. All values are shown as the mean ± standard deviation (SD). Statistical analyses were conducted with Student’s *t*-test.

## Results

### Generation of Lcn9-Cre KI mouse

**Fig 1:**
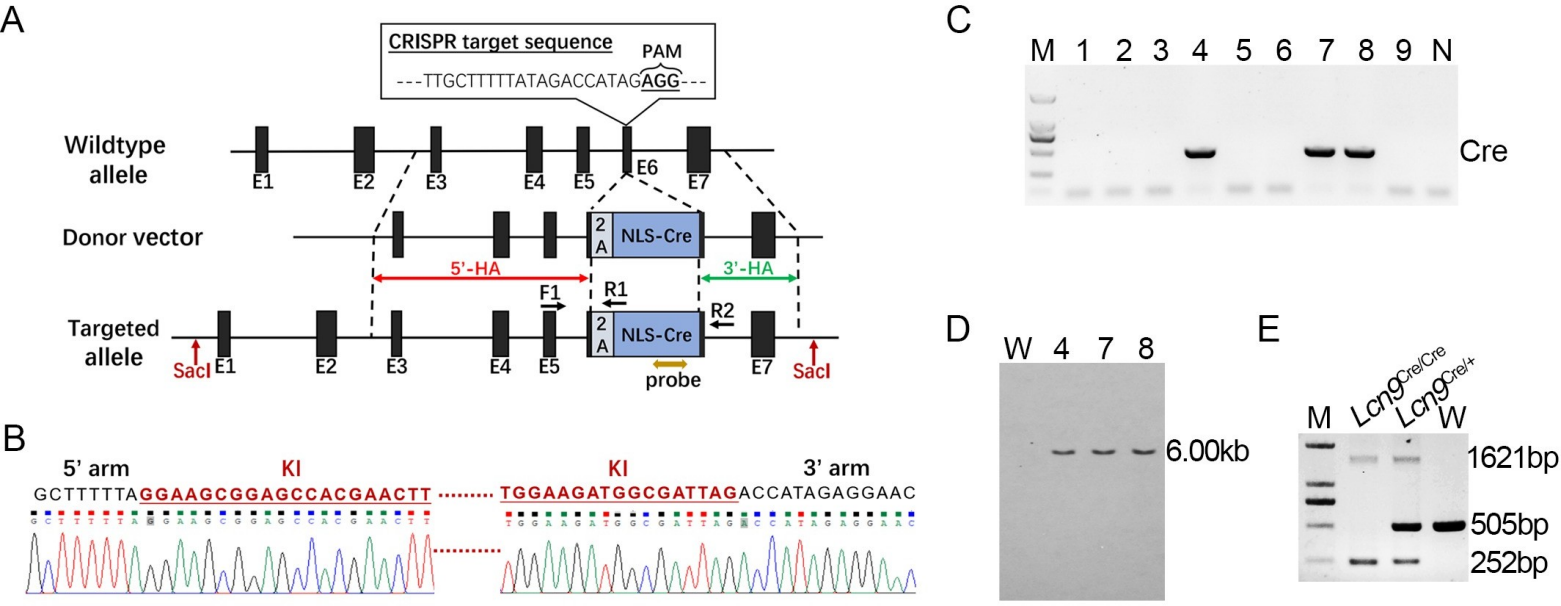
Generation of the Lcn9-Cre mouse line. (A) Schematic diagram of the knock-in strategy of the Lcn9-Cre mouse line. The sgRNA targeted exon 6 of mouse Lcn9. The TAG stop codon was replaced with the 2A-NLS-Cre cassette. The 1.0 kb 5’-homology arm and the 970 bp 3’-homology arm are marked with red and green double-headed arrows, respectively. Black arrows indicate the primer sets for genotyping. The brown double-headed arrow indicates the probe for Southern blotting. E, exon; HA, homology arm; NLS, nuclear localization signal; PAM, protospacer adjacent motif. (B) Genomic DNA sequence chromatograms of Lcn9-Cre KI mice showed the 5’ junction and 3’ junction of the insertion (inserted sequence marked in red). (C) PCR genotyping of 9 F1 pups derived from one Cre-positive founder using a pair of primers targeting the Cre insertion. Pups 4, 7 and 8 were Cre-positive. (D) Southern blotting analysis of SacI-digested tail DNA from Cre-positive mice. Predicted knock-in allele is 6.00 kb. W, wild-type control. (E) Typical genotyping of WT and Lcn9-Cre KI mice (1621 bp and 252 bp for KI allele, and 505 bp for WT allele) with primers F1/R1/R2.

The mouse *Lcn9* gene consists of 7 exons with the ATG start codon in exon 1 and the TAG stop codon in exon 6. To generate an allele in which Cre recombinase expression recapitulates endogenous Lcn9 expression without disrupting Lcn9 function, we integrated the 2A-NLS-Cre cassette into exon 6 at the TAG stop codon locus (Fig 1A). As a result, Cre expression was driven by the *Lcn9* promoter in tandem with endogenous Lcn9 expression as expected. The target insertion was amplified by PCR and confirmed with sequence analysis (Fig 1B). F1 mice 4, 7 and 8, derived from positive F0 mice, were identified with positive Cre insertion (Fig 1C). Correct Cre insertion into Lcn9 locus was also confirmed by Southern blotting. All positive Cre KI mice were detected with a 6.00 kb fragment (Fig 1D). The Cre KI pups were genotyped by PCR with primers F1/R1/R2. A 505 bp-band was assessed in WT offspring. A 252 bp-band and a 1621 bp-band were monitored in homozygotes (Fig 1E).

### Region-specific and temporal expression of Cre recombinase in Lcn9-Cre KI mice

**Fig 2:**
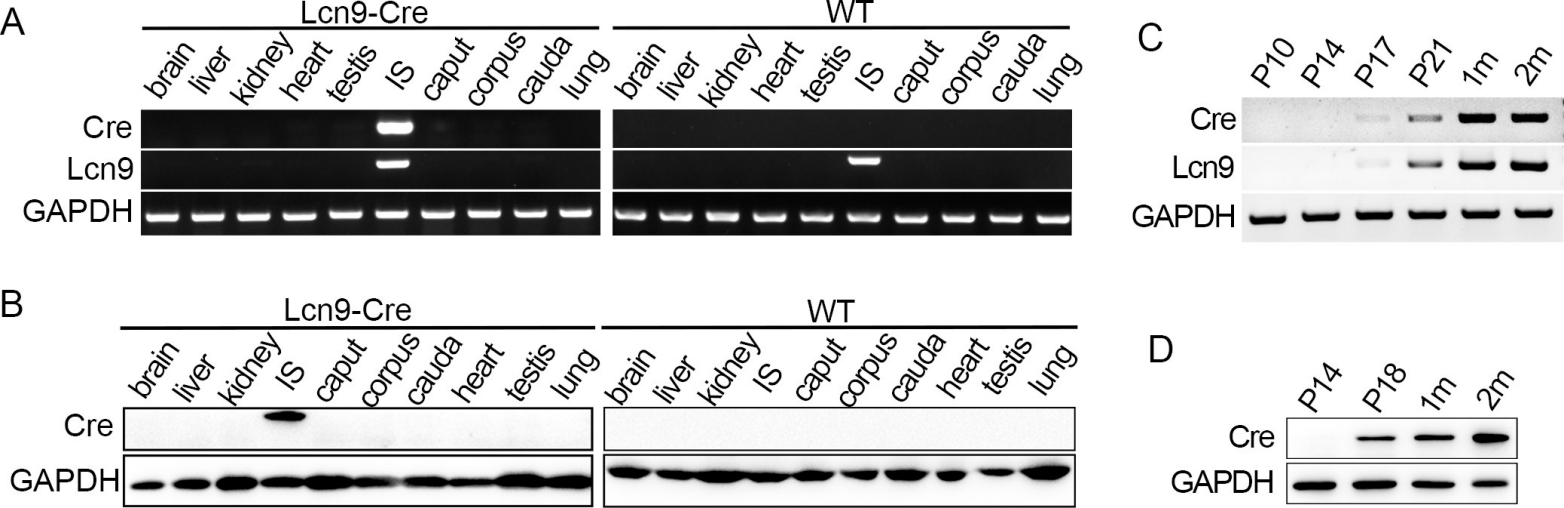
Spatial-temporal expression of Cre recombinase in Lcn9-Cre mice. (A) Tissue-specific mRNA distribution of Cre and endogenous *Lcn9* detected by RT-PCR in 2-month-old homozygous males and WT controls. (B) Western blotting analysis of Cre distribution in different tissues. (C) Changes of Cre recombinase and *Lcn9* in epididymal IS in postnatal development of Lcn9-Cre males at different ages were assessed by RT-PCR. (D) Western blotting analysis of Cre expression in epididymal IS of postnatal Lcn9-Cre males at different ages. P10, postnatal day 10; P14, postnatal day 14; P17, postnatal day 17; P18, postnatal day 18; P21, postnatal day 21; 1m, 1-month-old; 2m, 2-month-old. GAPDH served as the internal reference.

To check the spatial expression of Cre recombinase under the *Lcn9* promoter, tissues including brain, liver, kidney, heart, testis, lung, and four epididymal regions, were collected from 2-month-old WT and Cre KI mice. Cre mRNA expression was evaluated by RT-PCR. No band was detected in any of the tested tissues from the WT controls (Fig 2A). However, Cre mRNA was detectable in the IS of Lcn9-Cre mice, with a 151 bp band observed, indicating the IS-specific distribution of Cre (Fig 2A). Simultaneously, normal expression of *Lcn9* mRNA was examined to exclude the possibility of Cre insertion affecting original expression of *Lcn9* gene. Western blotting was performed to examine Cre protein expression. A 37 kDa single band was detected exclusively in the IS samples from Lcn9-Cre KI males (Fig 2B). Therefore, Cre recombinase is expressed specifically in the IS, driven by the endogenous *Lcn9* promoter.

The change in Cre mRNA and protein expression over time was examined by RT-PCR and Western blotting. RNA and protein samples were prepared from the epididymal IS of Lcn9-Cre KI homozygotes. Consistent with the expression changes of endogenous *Lcn9*, the initial mRNA expression of the Cre recombinase was detected at postnatal day 17, with a significantly high level detected at postnatal 2 months (Fig 2C). No Cre protein expression was observed in the samples extracted from Lcn9-Cre KI males at day 14, while detectable, elevated expression was observed from postnatal day 18 to 2 months (Fig 2D), consistent with the mRNA expression pattern.

### Identification of Cre-mediated DNA recombination with a reporter mouse line

**Fig 3:**
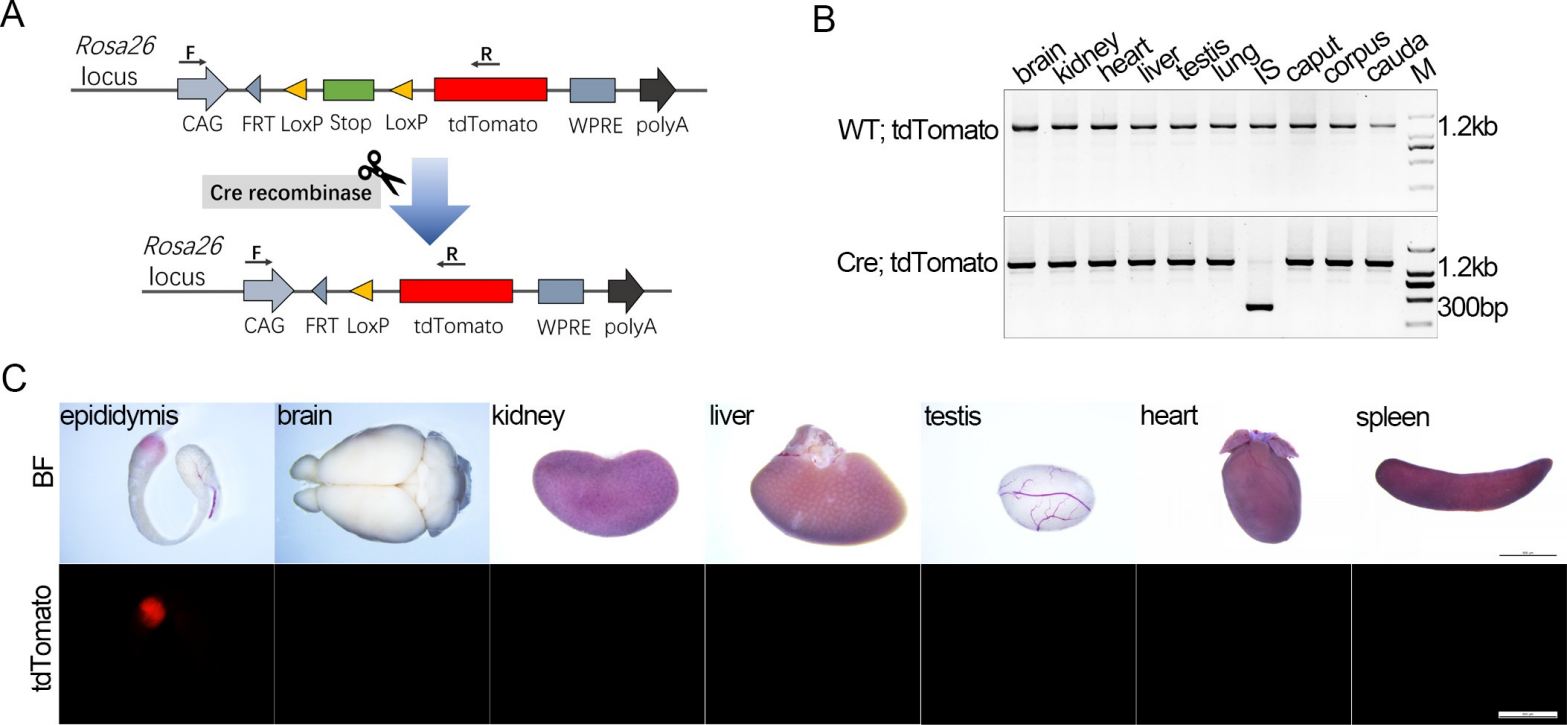
Spatial distribution of active Cre recombinase in Lcn9-Cre KI mice. (A) Schematic diagram of the Rosa26^tdTomato^ reporter mice used in the study and Cre recombinase-mediated activation of tdTomato expression. In Cre; Rosa26^tdTomato^ mice, Cre recombinase mediates the deletion of the stop sequence between the loxP sites, resulting in tdTomato expression in cells with Cre activity. Primers of F/R were used to identify Cre-mediated genomic DNA alteration. FRT, FLPP recombination target; WPRE, regulatory element of woodchuck hepatitis virus. (B) Detection of recombinant alleles in various tissues of Lcn9-Cre; Rosa26^tdTomato^ mice by PCR. A smaller 300 bp band of recombined allele was detected exclusively in the IS from Lcn9-Cre mice. (C) tdTomato signals detection in various tissues with Cre recombination, collected from 5-month-old Lcn9-Cre; Rosa26^tdTomato^ mice. Scale bar = 500 μm.

To detect Cre recombination, we crossed Cre KI mice with the Rosa26^tdTomato^ reporter mice, which possess two loxP sites on either side of a stop element preceding a tdTomato cassette. In Cre; Rosa26^tdTomato^ mice, Cre recombinase mediates deletion of the stop sequence between the loxP sites, resulting in tdTomato expression in cells with active Cre (Fig 3A).

To test Cre-mediated fragment deletion, a pair of primers flanking the loxP sites were designed to amplify the recombinant allele (Fig 3A and S1 Table). Ten types of tissues were isolated to extract genomic DNA and prepared for PCR amplification. No recombinant allele but a 1.2 kb product was detected in the WT; Rosa26^tdTomato^ control (Fig 3B). In Lcn9-Cre; Rosa26^tdTomato^ mice, a smaller 300 bp recombinant allele was observed only in the IS sample, indicating the IS-specific Cre-mediated recombination.

To screen for spatial distribution of Cre activity, different tissues including epididymis, brain, kidney, liver, testis, heart and spleen, were collected from Lcn9-Cre; Rosa26^tdTomato^ mice and examined with a fluorescence stereomicroscope. tdTomato expression was detected in the epididymis, predominantly restricted to the IS, while no signal was observed in other tissues (Fig 3C). Thus, Lcn9-Cre KI mice exhibited IS-specific Cre activity.

**Fig 4:**
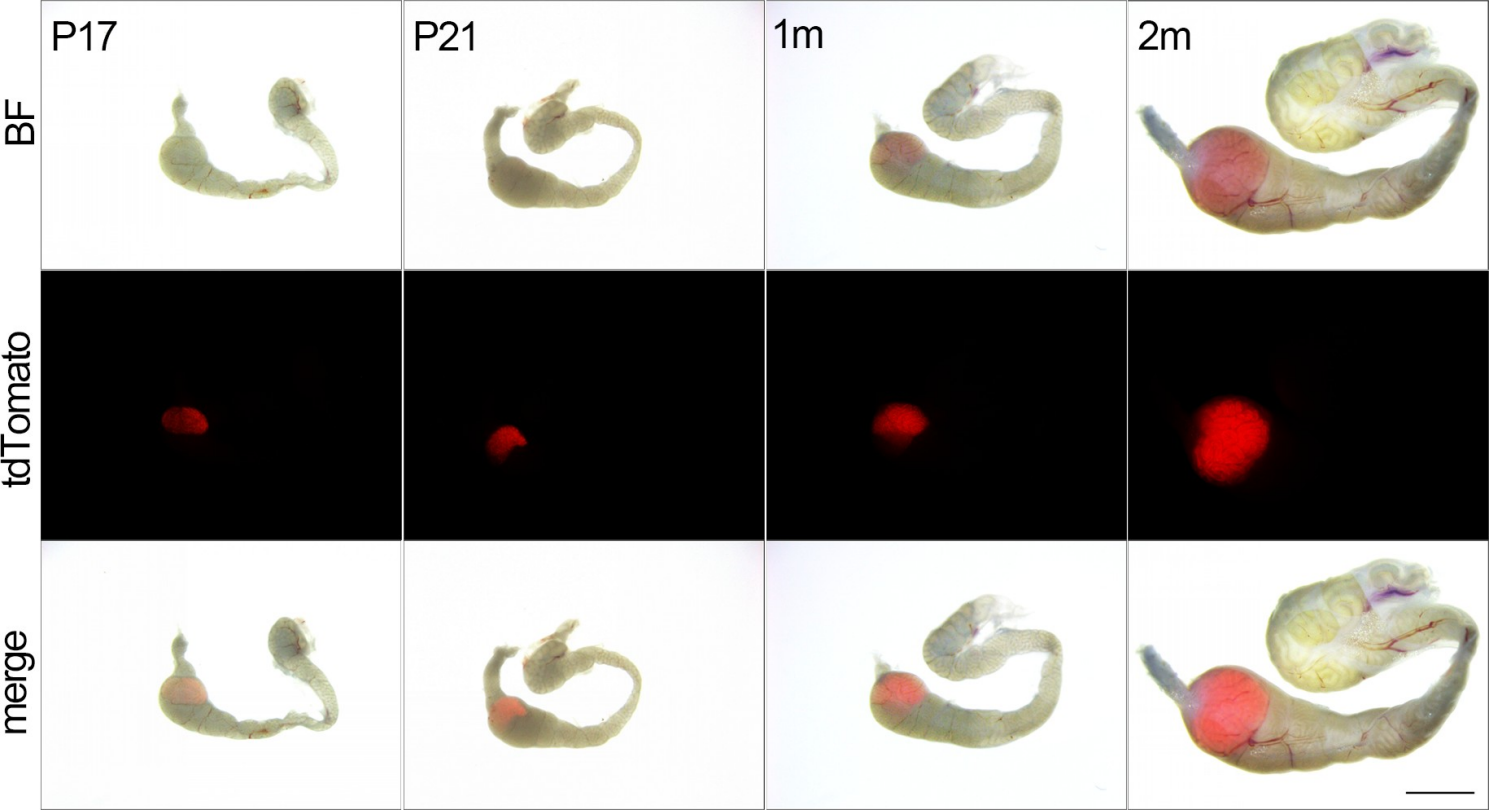
Temporal distribution of active Cre recombinase in Lcn9-Cre mice. tdTomato signals detected in epididymides collected from Lcn9-Cre; Rosa26^tdTomato^ males at different postnatal stages from P17 to 2m. Scale bar = 2 mm. BF, bright field.

Time course analyses of Cre activity were conducted by determining the tdTomato signals in postnatal development of IS from Cre; Rosa26^tdTomato^ males. tdTomato expression in the IS was initially observed on P17, and it continued to increase during the first 2 months, driven by the *Lcn9* promoter, consistent with the Cre mRNA and protein expression patterns (Fig 4).

**Fig 5:**
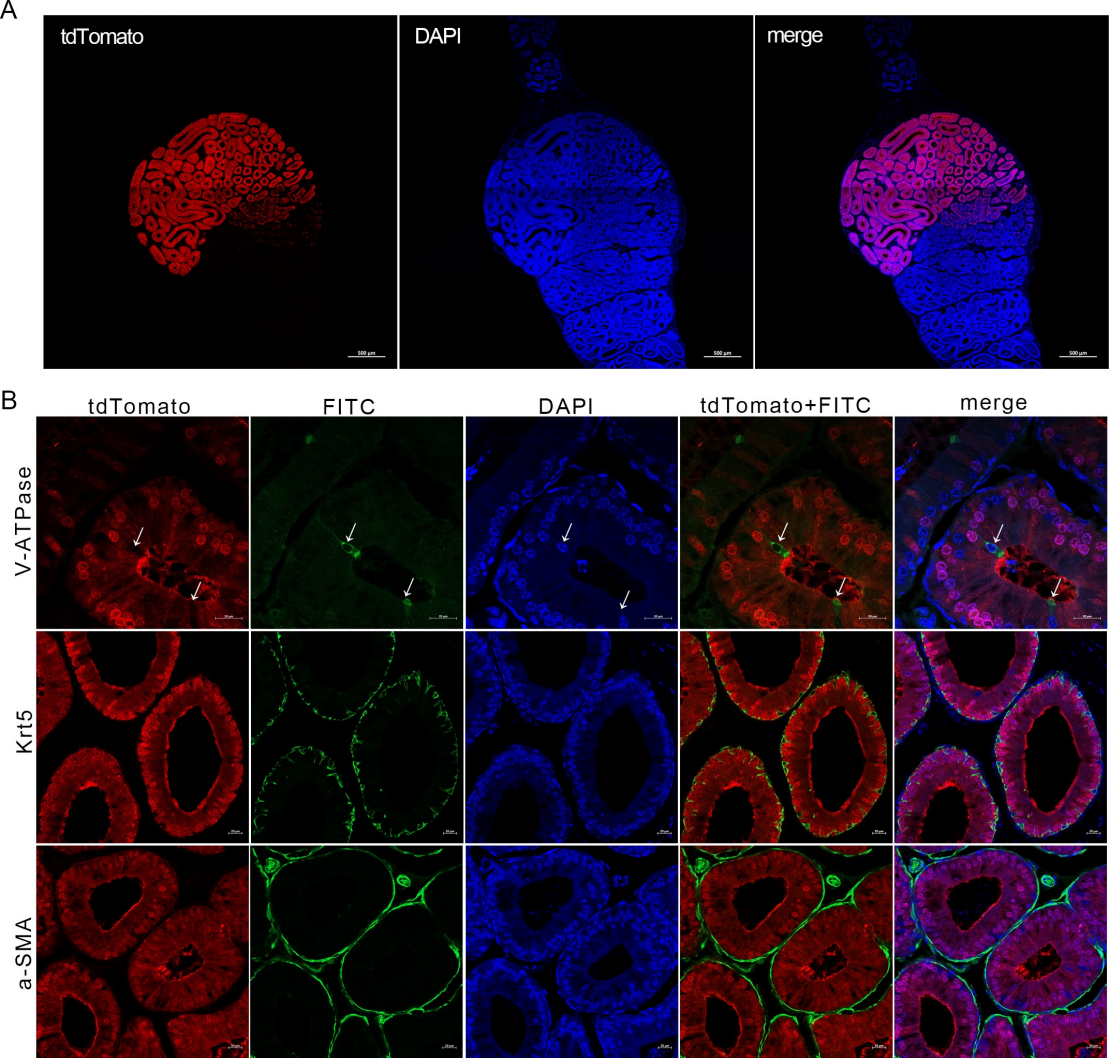
Epididymal region-specific and cellular localization of active Cre recombinase in Lcn9-Cre mice. (A) The proximal region-specific distribution of tdTomato signals detected in epididymis with Cre recombination, collected from 2-month-old Lcn9-Cre; Rosa26^tdTomato^ males. (B) Cellular distribution of tdTomato signals detected in IS epididymal epithelium with Cre recombination, collected from 2-month-old Lcn9-Cre; Rosa26^tdTomato^ males. V-ATPase, a marker for narrow cells; Krt5, a marker for basal cells; a-SMA, a marker for smooth muscle cells. FITC indicates the signals labelled by antibodies. Nuclei were visualized with DAPI. White arrows indicate the narrow cells labelled by V-ATPase without tdTomato signals. (A) Scale bar = 500 μm; (B) Scale bar = 20 μm.

Epididymal tissues were also collected from adult Lcn9-Cre; Rosa26^tdTomato^ males and prepared as cryosections to examine tdTomato distribution in more detail. tdTomato fluorescence, indicating the Cre recombination, was observed throughout the entire IS (Fig 5A). No detectable expression was monitored in the efferent duct or other epididymal regions. Consistent with the information from previous studies [26], a mosaic distribution of tdTomato signals was observed at the boundary between the IS and proximal caput.

### Identification of cell types with Cre activity in Lcn9-Cre mouse

Given the fact that the epithelial duct of IS consists mainly of principal cells, narrow cells, smooth muscle cells and basal cells, the cell types with Cre activity were identified with immunostaining of cryosections from Lcn9-Cre; Rosa26^tdTomato^ epididymal samples. As shown in Fig 5B (white arrows), narrow cells labeled by V-ATPase, elongated in the direction of apical to basal axis and contacted the epididymal lumen with their apical surface; there was no colocalization with tdTomato positive cells. Basal cells, identified specifically by Krt5 antibody, were located at the base of the epididymal epithelium and presented lateral body projections toward the lumen, and no double-positive cell was observed (Fig 5B). In addition, a-SMA-positive smooth muscle cells located lining the peripheral boundary of the epididymal ducts, were not included in the tdTomato positive position (Fig 5B). Unfortunately, even after testing a series of potential products, we could not obtain any effective commercial antibodies that specifically labeled the principal cells. However, considering the cell-type composition of the IS epithelial duct and the fact that Lcn9 is a secretory protein synthesized by principal cells, it could be inferred that the Cre activity was present exclusively in the principal cells lining the IS epithelium. Thus, the present results were consistent with previous reports of specific localization of Lcn9 in the principal cells of IS epithelium [26].

### Cre-mediated DNA recombination was further identified by crossing with *Tsc1* floxed alleles

**Fig 6:**
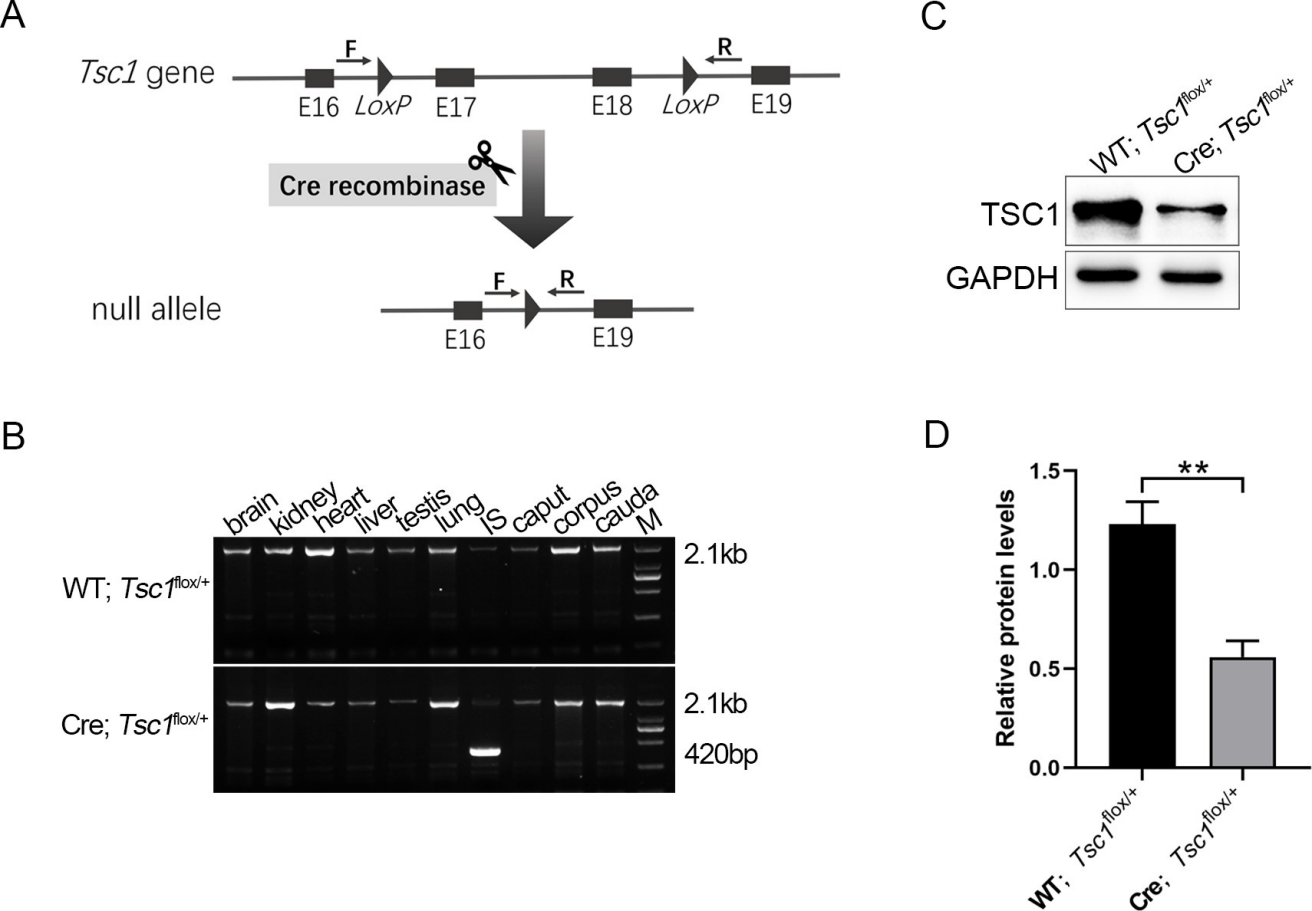
Cre activity *in vivo* confirmed by using Lcn9-Cre; *Tsc1*^flox/+^ mice. (A) Schematic diagram of the *Tsc1* floxed mice and Cre recombinase-mediated DNA deletion *in vivo*. Cre recombinase mediates the deletion of exons 17 and 18 between the loxP sites. F/R indicate the primer set for detecting Cre-mediated genomic DNA alteration. E, exon. (B) Detection of recombinant alleles in various tissues of *Tsc1*^flox/+^ mice by PCR. The WT band was 2.1 kb. A smaller 420 bp band of recombined allele was detected exclusively in the IS from Lcn9-Cre; *Tsc1*^flox/+^ mice. (C) Western blotting analysis to measure expressions of TSC1 protein in the IS, collected from 2-month-old males. (D) Quantification of TSC1 protein amount using ImageJ. (n = 3, **p<0.01).

To further check Cre activity *in vivo*, Lcn9-Cre; *Tsc1*^flox/+^ mice were generated by mating the Lcn9-Cre mouse strain with the *Tsc1*^flox/+^ mouse line. Cre recombinase mediates deletion of exons 17 and 18 between the two *loxP* sites, resulting in conditional knockout of *Tsc1* in cells with Cre activity. A 2.1 kb WT band was detected in the DNA from Lcn9-Cre; *Tsc1*^flox/+^ males and WT; *Tsc1*^flox/+^ controls, while a smaller 420 bp DNA fragment corresponding to the recombinant *Tsc1* allele was specifically observed in the IS samples from Lcn9-Cre; *Tsc1*^flox/+^ males (Fig 6A and 6B). The IS samples were collected from 2-month-old males, and the expression of TSC1 was tested through Western blotting. The amount of TSC1 protein decreased in the Lcn9-Cre; *Tsc1*^flox/+^ samples compared with WT; *Tsc1*^flox/+^ controls (Fig 6C and 6D). Thus, the Cre recombinase in the Lcn9-Cre KI mice can mediate the disruption of floxed genes *in vivo* exclusively in the epidydimal IS.

### The Cre KI mice are characterized by normal development

To determine whether Cre insertion and expression *in vivo* can induce an abnormal developmental phenotype or infertility, homozygous Lcn9-Cre KI mice were analyzed.

**Fig 7:**
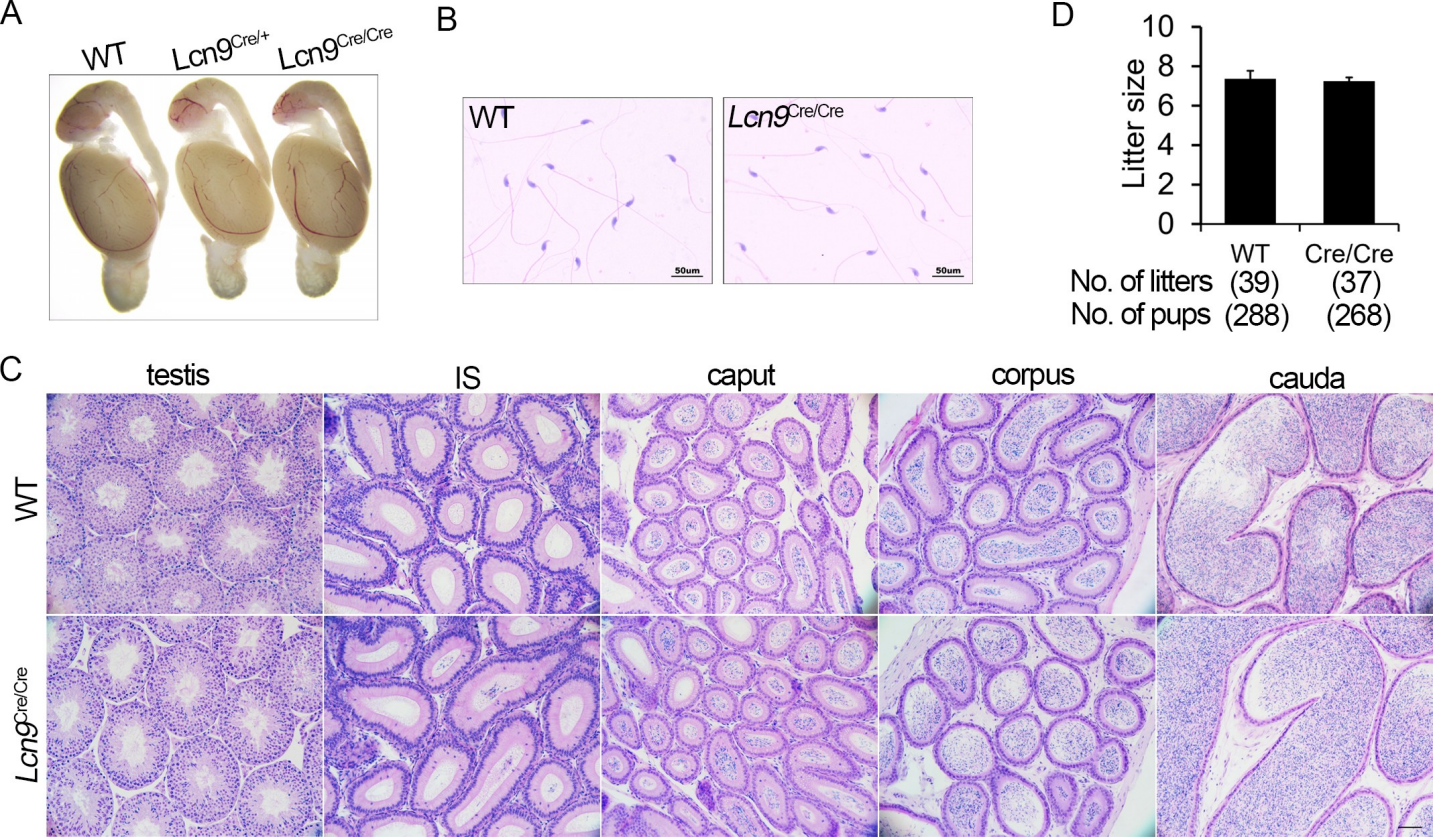
Fecundity of Lcn9-Cre KI mice. (A) Morphological images of male reproductive ducts from 6-month-old Lcn9-Cre KI males and WT controls. (B) Morphological observation of sperm collected from the cauda. (C) Histological analysis with H&E staining of the testes and epididymides collected from homozygous Cre KI males and WT controls. (D) Male fecundity. Comparable average litter size was observed between homozygous Cre KI males and WT controls (*p* = 0.62). (B) Scale bar = 50 μm; (C) Scale bar = 100 μm.

Firstly, we examined the behavior and appearance of homozygous mice, and observed no apparent abnormalities (data not shown). Comparison of morphology of tissues from postnatal homozygotes with those from WT controls indicated a normal developmental phenotype in homozygous mice (data not shown). Histological analyses showed an apparently normal morphological phenotype of testes and epididymides in Lcn9^Cre/Cre^ males and WT controls (Fig 7A and 7C). The cauda lumen was filled with high concentrations of sperm (Fig 7C). All homozygous males produced sperm with normal morphology (Fig 7B).

To determine whether Cre insertion induced abnormal fertility, two-month-old homozygous Cre KI males and WT controls were mated with C57BL/6J females. As is shown in Fig 7D, homozygous Lcn9-Cre males showed normal fecundity (average litter size: 7.2 ± 0.2 for Lcn9^Cre/Cre^, 7.4 ± 0.4 for WT controls; *p* = 0.62).

Taken together, the insertion and expression of Cre recombinase did not affect the normal development or male fertility.

## Discussion

In this study, a novel Cre KI mouse line with epididymal IS-specific Cre activity was generated via the CRISPR/Cas9 system. In this Cre mouse line, a 2A-NLS-Cre element was introduced into the endogenous *Lcn9* gene locus, ensuring that the Cre expression driven by the *Lcn9* promoter is in tandem with Lcn9 expression. The temporal and spatial expression of Cre mRNA was consistent with that of endogenous *Lcn9* in Lcn9-Cre mice according to the results of RT-PCR. The spatial expression patterns of Cre recombinase including IS- and principal cell-specific localization, were similar to those of endogenous Lcn9 reported previously [26, 27, 38].

Surprisingly, the temporal expression patterns of the Cre recombinase were not exactly identical with that previously reported for endogenous Lcn9. According to Northern blotting analysis, Lcn9 was not detectable before postnatal 3 weeks, correlated with the absence of testicular fluid circulating within the epididymal lumen [26]. However, in our Lcn9-Cre KI mouse lines, initial expression of Cre with normal recombinase activity could be detected on postnatal day 17 by RT-PCR or tdTomato observation within Lcn9-Cre; Rosa26^tdTomato^ mice. This discrepancy could be attributed to the sensitivity of the methods used in these studies to detect *Lcn9* mRNA expression, since the RT-PCR method is characterized by exponential amplification and is able to detect very low level of target molecules. Additionally, to detect the ontogenic expression of *Lcn9* mRNA, the epididymal samples were collected at weekly intervals in previous studies [26], while here the expression of Cre or Lcn9 was measured day by day from postnatal day 14 to day 21, in order to determine the time at which the genes were first expressed. The expression of *Lcn9* or Cre mRNA was quite weak on P17. And accordingly, a faint, mosaic pattern of tdTomato expression was observed in epididymal IS of Lcn9-Cre; Rosa26^tdTomato^ males of the same age, indicating only a few cells have positive Cre activity. For the temporal inconsistence, the other possibility might be the differences in the genetic background of the mice used in these studies or diet differences, which could potentially affect the growth rates of the mice.

In murine epididymis, as an evident portion of the proximal region, the IS is considered to be responsible for the absorption of the fluid from the rete testis, which is necessary for sperm concentration, and functions as a major player in spermatozoa maturation [7–9, 14]. Principal cells, which account for the majority of the epididymal epithelium, are highly active absorptive and secretory cells responsive for the synthesis of almost all proteins secreted into the epididymal lumen [39]. Within principal cells, gene expression and protein secretion are tightly regulated and region-dependent, and the compartmentalized gene expression contributes to region-specific luminal protein profile and segment-restricted microenvironment for spermatozoa maturation [17, 40]. The identification and elucidation of the functions of genes expressed in the principal cells originating in IS region, remains an ongoing effort. Up to now, several epididymis-specific Cre mouse models have been reported with Cre activity in the epididymal IS region, but none exhibited Cre expression limited exclusively to the entire IS region. In Crisp4 iCre KI mice, the distribution of active Cre was monitored in the IS, caput, corpus and cauda with different levels found in each region [25], while in the Defb41 iCre mouse line, Cre expression was examined in both the IS and caput [23]. In Rnase10-Cre mice, Cre activity was limited to only a part of the epididymal IS, an expression pattern identical to that of Rnase10 itself [24, 41]. Therefore, with the expression of Cre recombinase specific to principal cells throughout the entire IS, the Lcn9-Cre mouse line should be an appropriate tool for functional studies of genes in the epididymal IS using the Cre/*loxP* system.

## Supporting information

S1 Table Primers used in the present study. (DOCX)

S1 Raw images (PDF)
