# Supplementary material for: A novel mouse line with epididymal initial segment-specific expression of Cre recombinase driven by the endogenous Lcn9 promoter
Source: PLoS One. 2021 Jul 26;16(7):e0254802. doi: 10.1371/journal.pone.0254802 (PMC8312960; doi:10.1371/journal.pone.0254802)
Supplement: S1 Table — (DOCX) [file pone.0254802.s001.docx]

**S1 Table Primers used in the present study**

| **Primer name** | **Sequence (5’ to 3’)** | **Description** |
| --- | --- | --- |
| Lcn9-sgRNA-F | CACCTTGCTTTTTATAGACCATAG | To generate Lcn9 target sequence |
| Lcn9-sgRNA-R | AAACCTATGGTCTATAAAAAGCAA |  |
| Cre-common-F | GCCTGCATTACCGGTCGATGC | For F1 Cre mice genotyping or Cre mRNA detection |
| Cre-common-R | CAGGGTGTTATAAGCAATCCC |  |
| KI-probe-F | GGTCGATGCAACGAGTGATGAG | To amplify the probe template for Southern blotting |
| KI-probe-R | GAACGAACCTGGTCGAAATCAG |  |
| Lcn9-Cre-F1 | CTGCAATCTATATGGGCTGGACTC | For Lcn9-Cre mice genotyping |
| Lcn9-Cre-R1 | TTTTCTTCAACATCTCCTGCTTGC |  |
| Lcn9-Cre-R2 | CAGGGTAGTTTCCCACTCTTCTCAG |  |
| Tsc1-F | GTCACGACCGTAGGAGAAGC | For *Tsc1*^flox^ mice genotyping |
| Tsc1-R | GAATCAACCCCACAGAGCAT |  |
| Cre-F | GCCTGCATTACCGGTCGATGC | For Cre mRNA detection |
| Cre-R | CAGGGTGTTATAAGCAATCCC |  |
| Lcn9-F | ATGGTACTACTACTAGTCCTG | For Lcn9 mRNA detection |
| Lcn9-F1 | ACAACCTGGCCAGGATTTCC |  |
| Lcn9-R | TGTGAGTCCAGCCCATATAG |  |
| GAPDH-F | TGTTTGTGATGGGTGTGAACCA | For GAPDH mRNA detection |
| GAPDH-R | ACCAGTGGATGCAGGGATGATG |  |
| Tomato-WT-F | AAGGGAGCTGCAGTGGAGTA | For Rosa26^tdTomato^ mice genotyping |
| Tomato-WT-R | CCGAAAATCTGTGGGAAGTC |  |
| Tomato-mut-F | GGCATTAAAGCAGCGTATCC |  |
| Tomato-mut-R | CTGTTCCTGTACGGCATGG |  |
| tdTomato-RC-F | AGCTCCTGGGCAACGTGCTG | To detect the recombined alleles mediated by Cre recombinase |
| tdTomato-RC-R | TTGGTCACCTTCAGCTTGGCG |  |
| Tsc1-RC-F | AGGAGGCCTCTTCTGCTACC | To detect the recombined *Tsc1* floxed alleles mediated by Cre recombinase |
