# Supplementary material for: A novel mouse line with epididymal initial segment-specific expression of Cre recombinase driven by the endogenous Lcn9 promoter
Source: PLoS One. 2021 Jul 26;16(7):e0254802. doi: 10.1371/journal.pone.0254802 (PMC8312960; doi:10.1371/journal.pone.0254802)
Supplement: S1 Raw images — (PDF) [file pone.0254802.s002.pdf]

Fig 1C

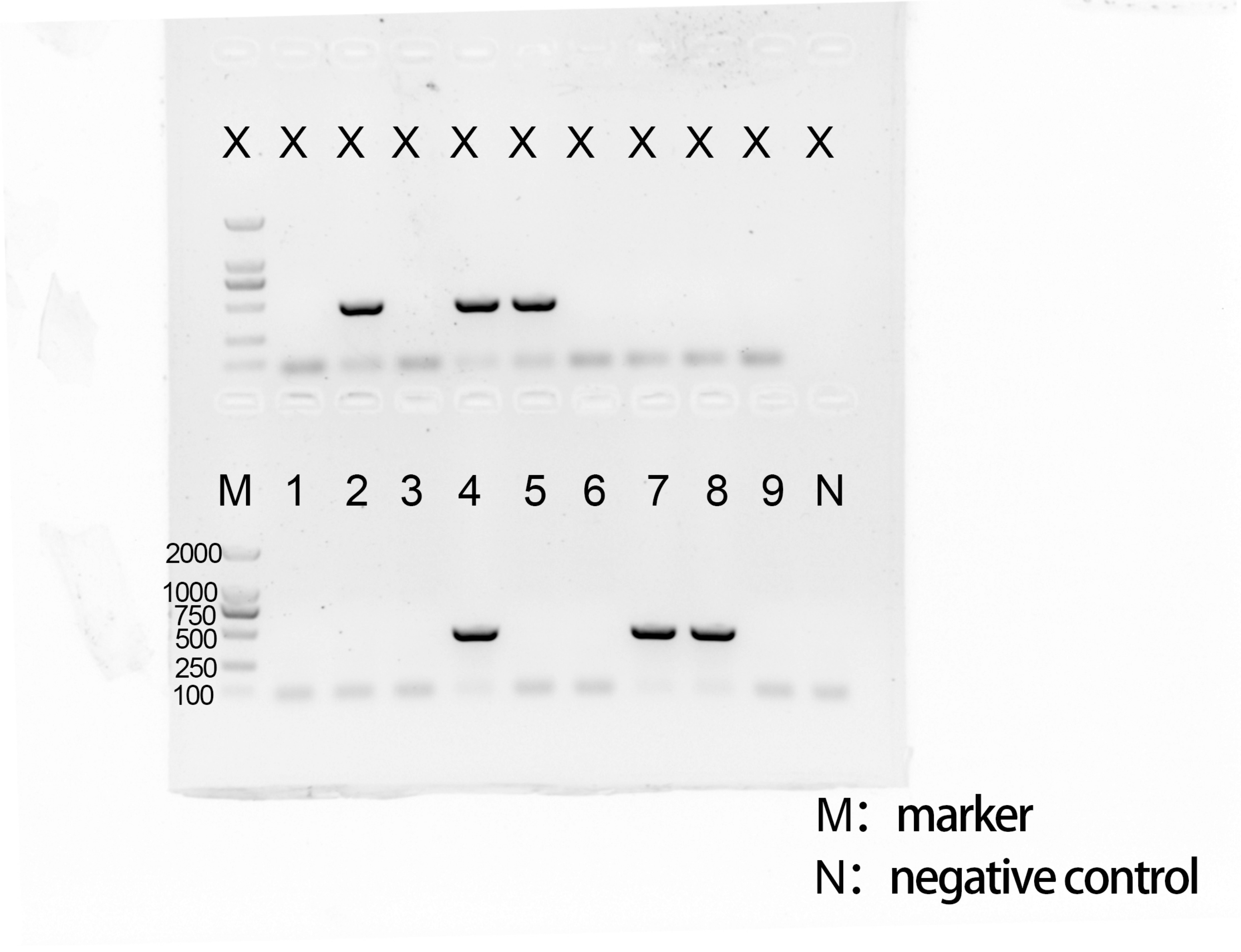
X X X X X X X X X X X
M 1 2 3 4 5 6 7 8 9 N
2000
1000
750
500
250
100
M: marker
N: negative control

Fig 1D

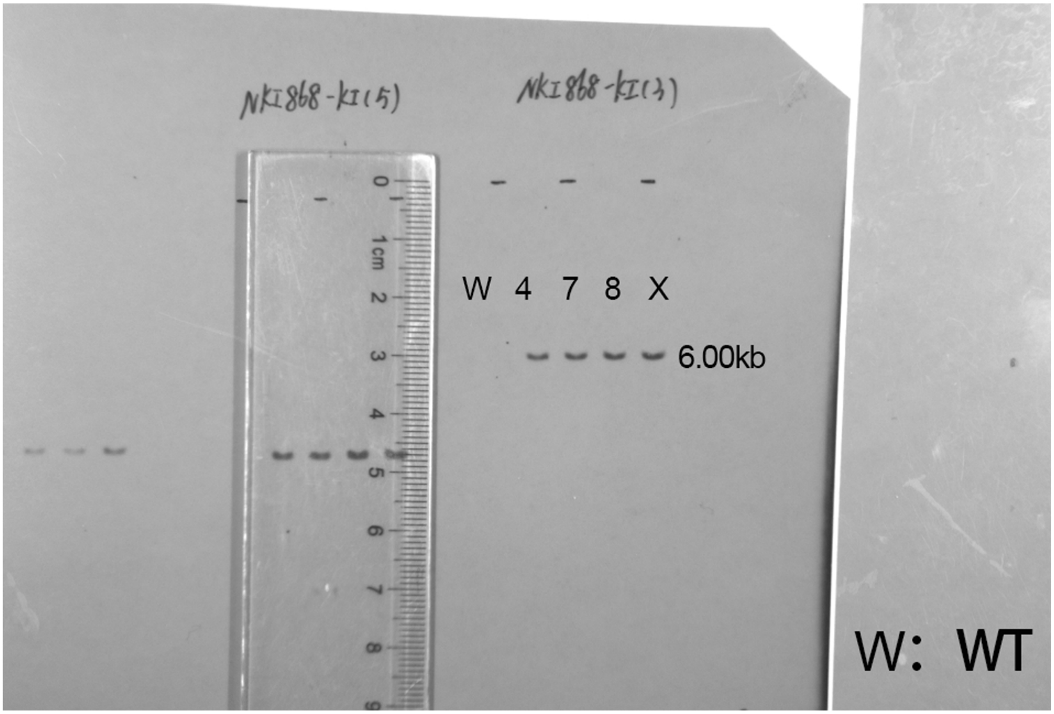

Fig 1E

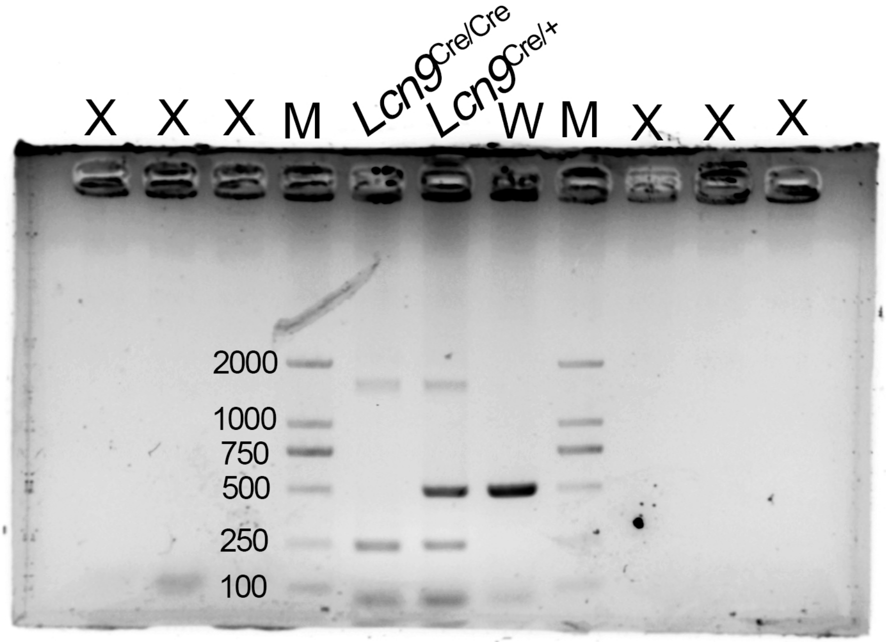

M： marker
W： WT

Fig 2A

Fig 2A Cre

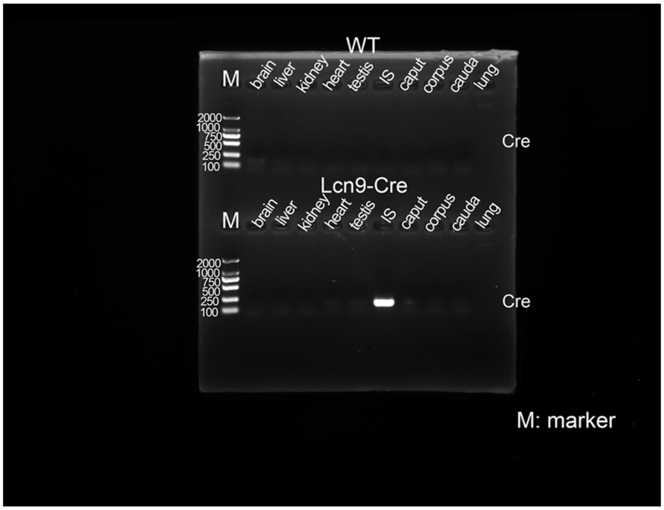

Fig 2A Lcn9

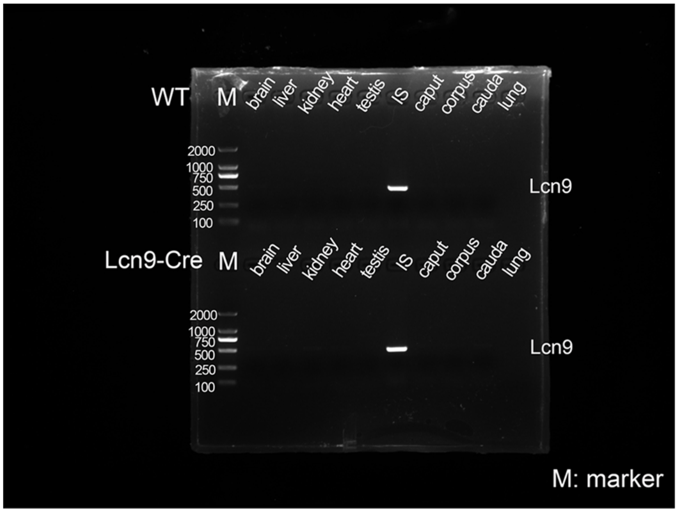

Fig 2A GAPDH for Lcn9-Cre samples

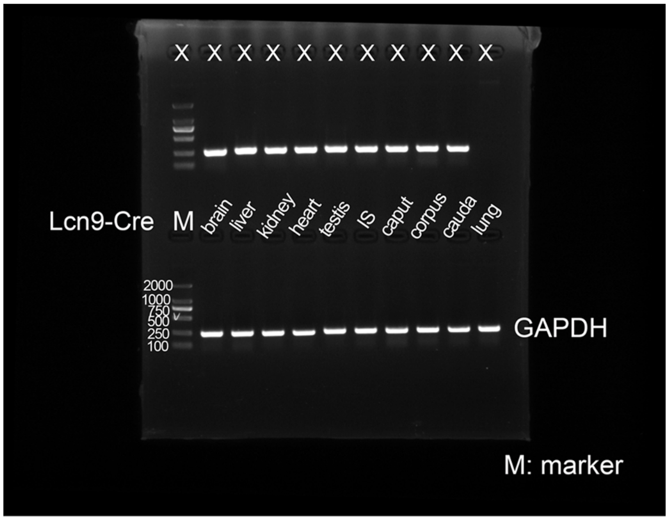

Fig 2A GAPDH for WT samples

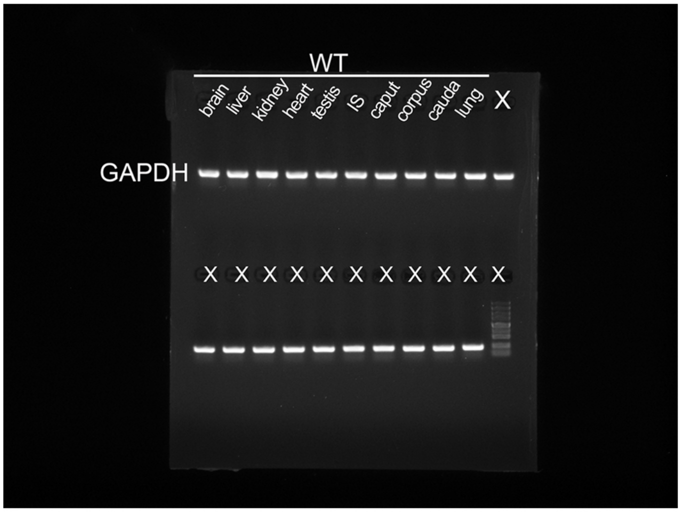

Fig 2B

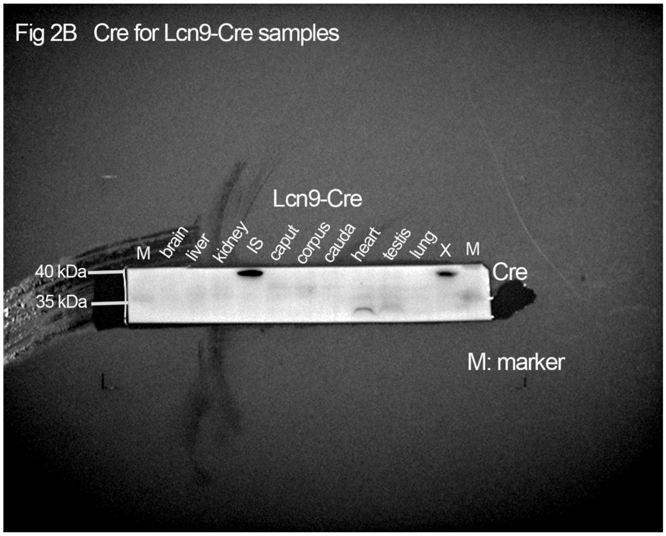

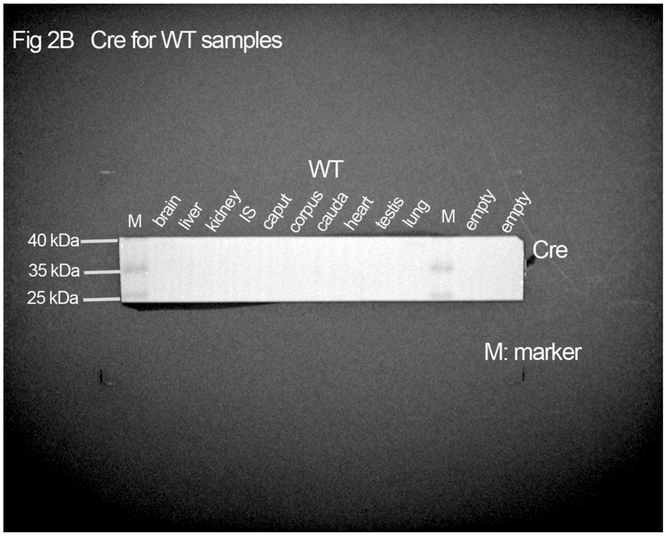

Fig 2B GAPDH for Lcn9-Cre samples

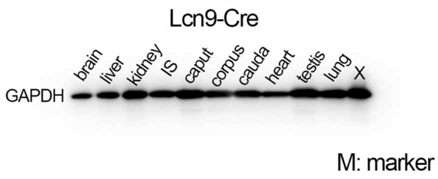

Fig 2B GAPDH for WT samples

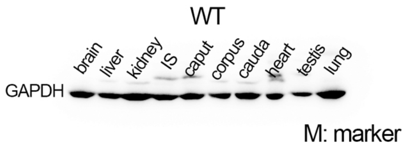

Fig 2C

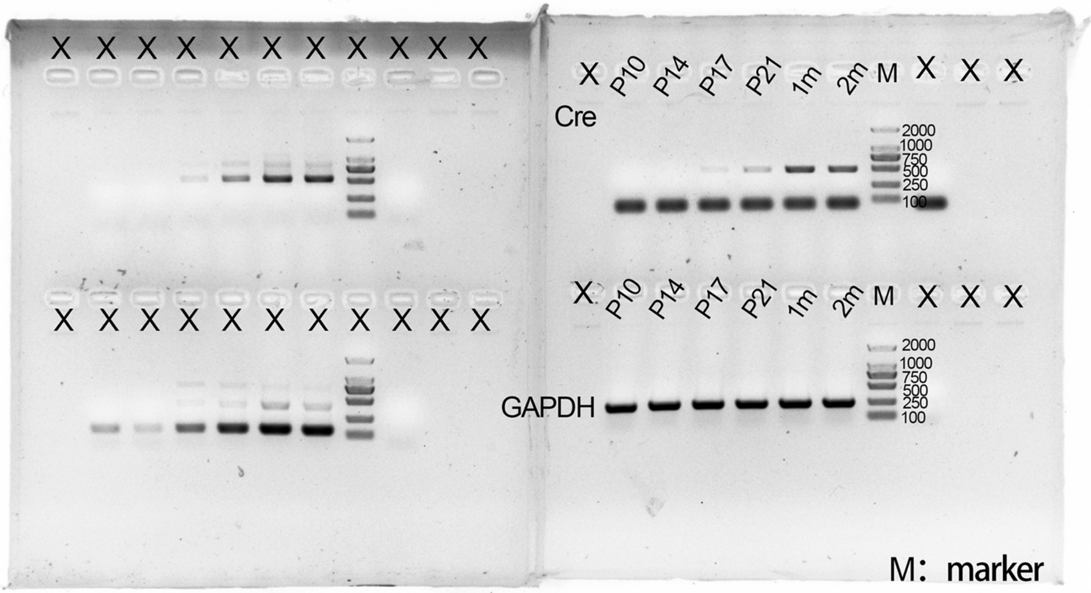
X X X X X X X X X X X
X X X X X X X X X X X
X P10 P14 P17 P21 1m 2m M X X X
Cre
2000
1000
750
500
250
100
X P10 P14 P17 P21 1m 2m M X X X
GAPDH
2000
1000
750
500
250
100
M: marker

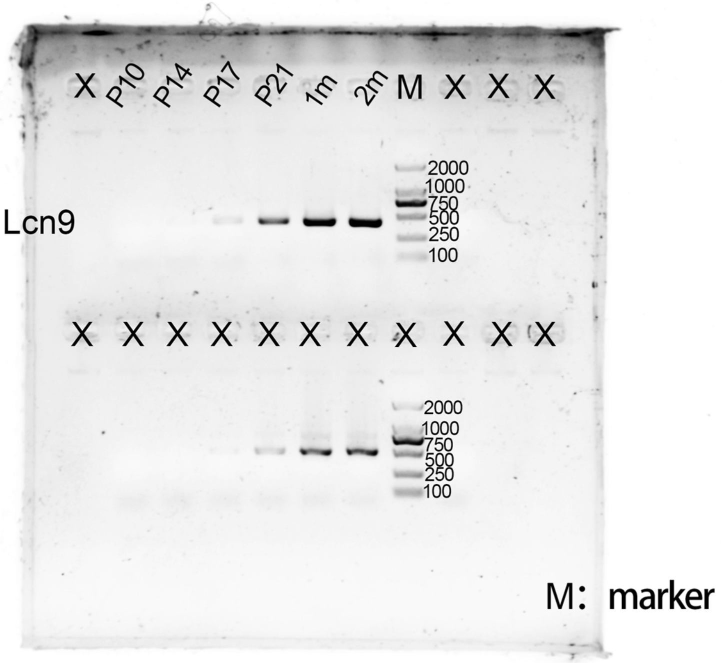
X P10 P14 P17 P21 1m 2m M X X X
Lcn9
2000
1000
750
500
250
100
X X X X X X X X X X X
2000
1000
750
500
250
100
M: marker

Fig 2D

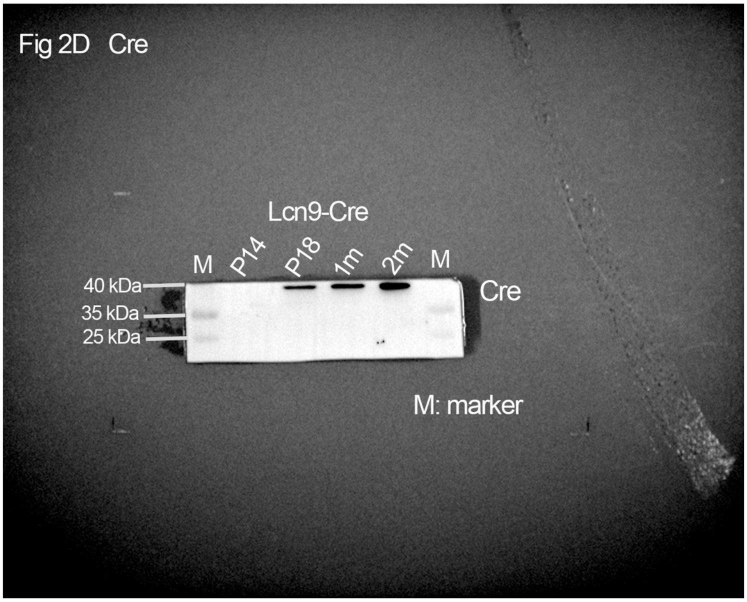
Fig 2D Cre
Lcn9-Cre
M
P14
P18
1m
2m
M
40 kDa
35 kDa
25 kDa
Cre
M: marker

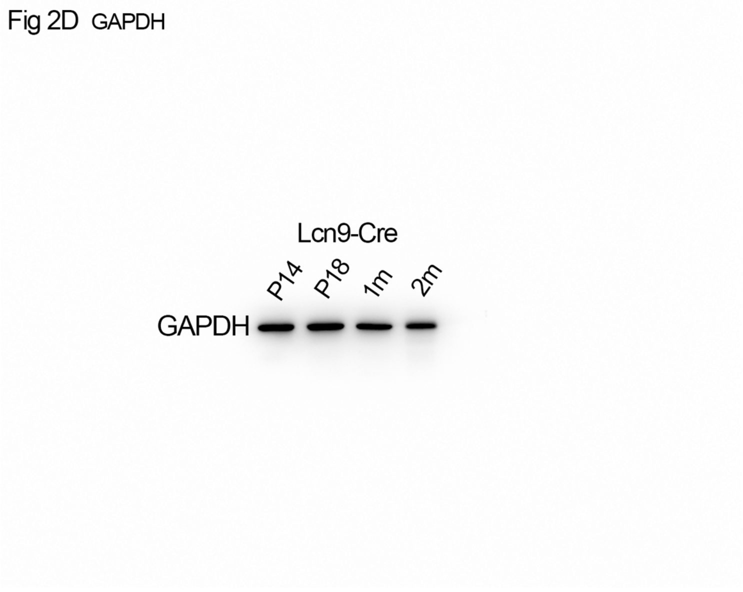
Fig 2D GAPDH
Lcn9-Cre
P14
P18
1m
2m
GAPDH

Fig 3B WT

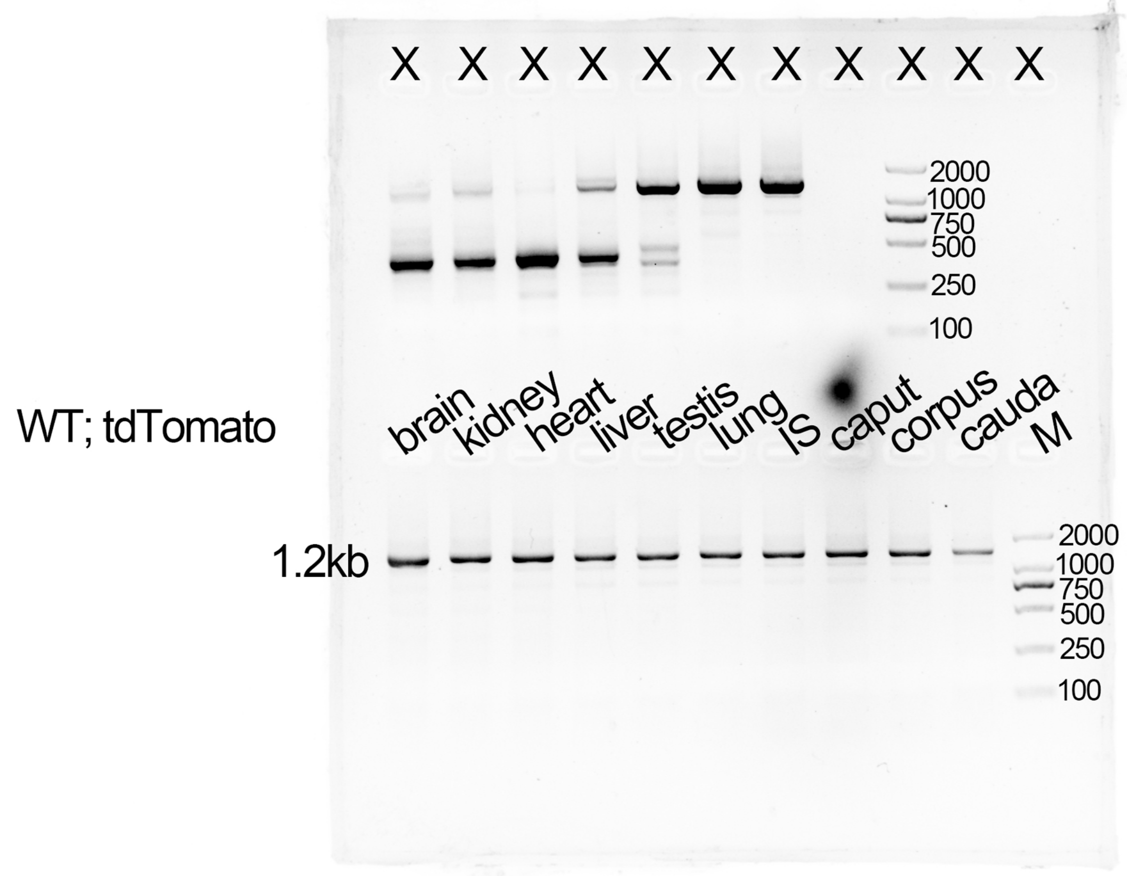

M：marker

Fig 3B Cre

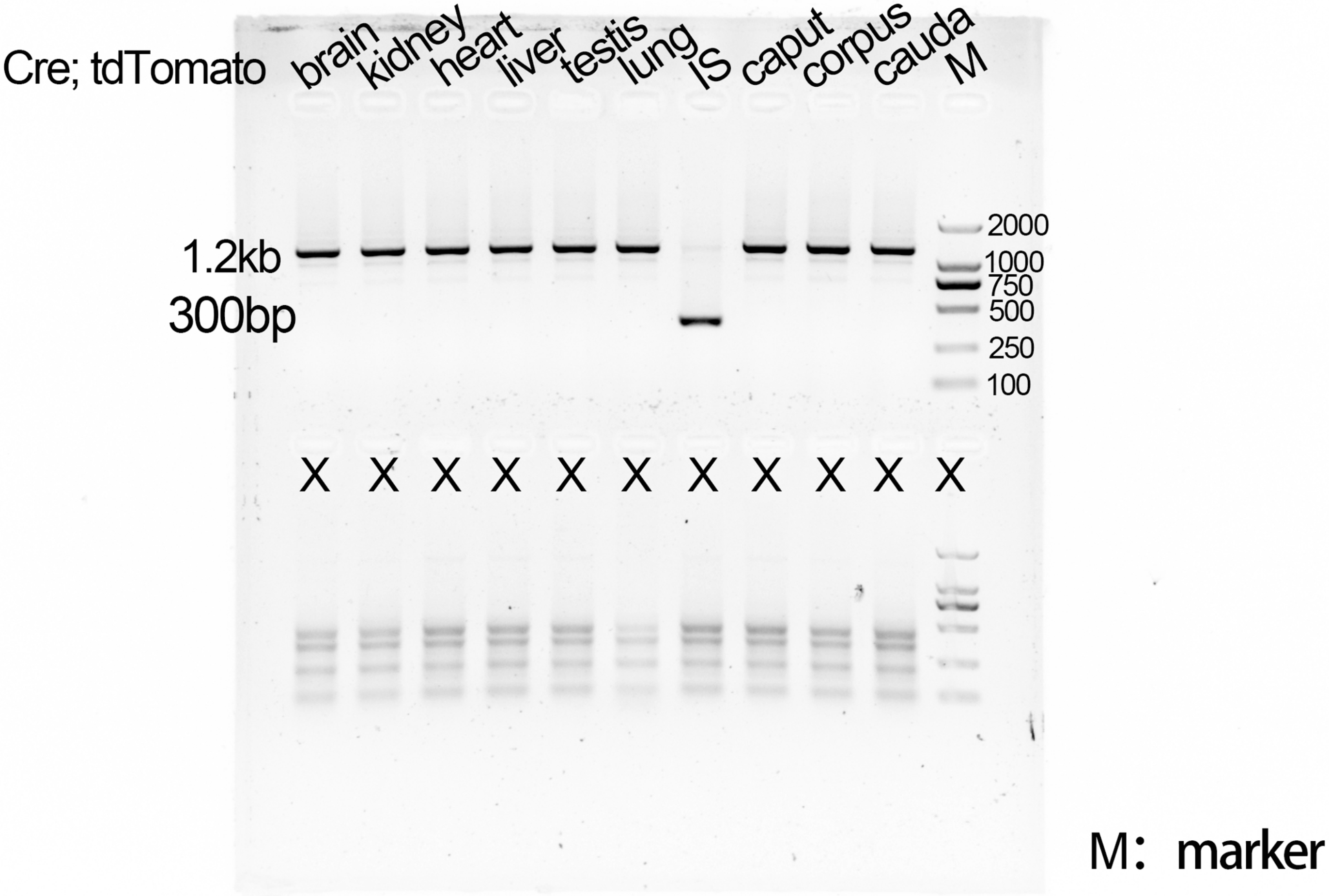

Cre; tdTomato
brain
kidney
heart
liver
testis
lung
IS
caput
corpus
cauda
M
1.2kb
300bp
2000
1000
750
500
250
100
X X X X X X X X X X X
M: marker

Fig 6B

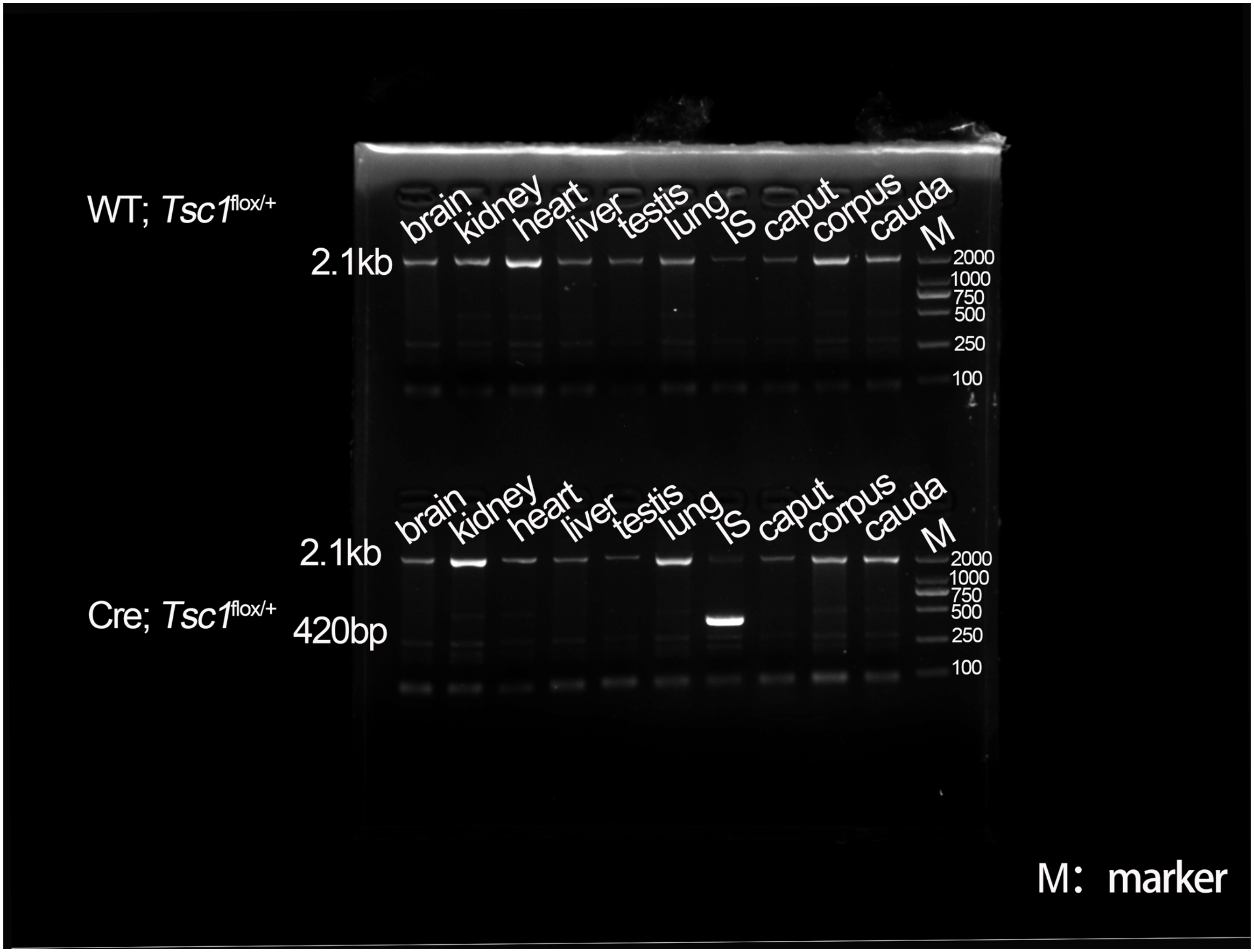
WT; Tsc1flox/+
brain
kidney
heart
liver
testis
lung
IS
caput
corpus
cauda
M
2.1kb
2000
1000
750
500
250
100
Cre; Tsc1flox/+
2.1kb
420bp
M: marker

Fig 6C

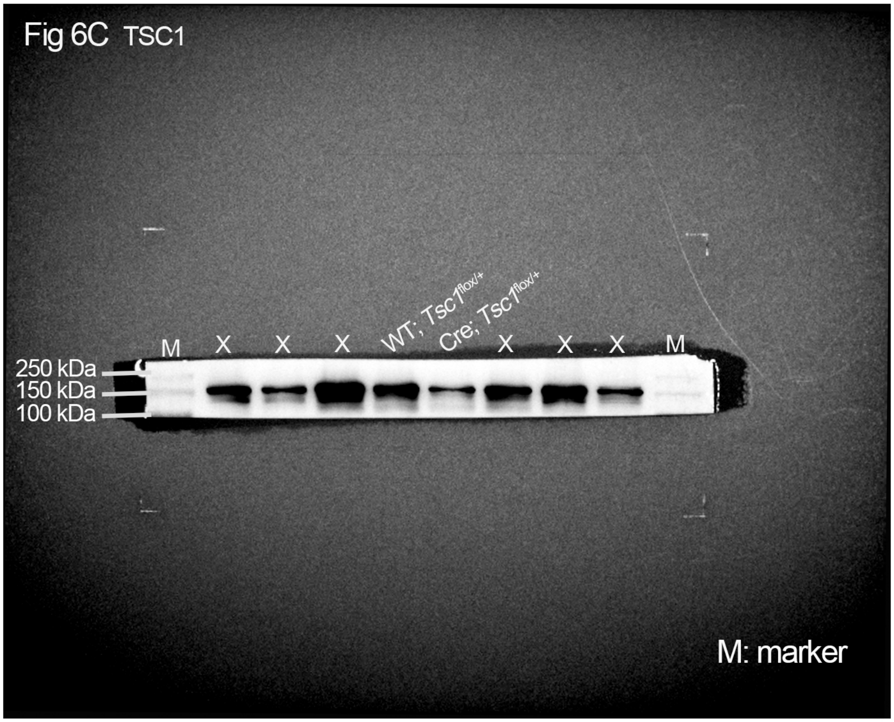
Fig 6C TSC1
M
X
X
X
WT; Tsc1flox/+
Cre; Tsc1flox/+
X
X
X
M
250 kDa
150 kDa
100 kDa
M: marker

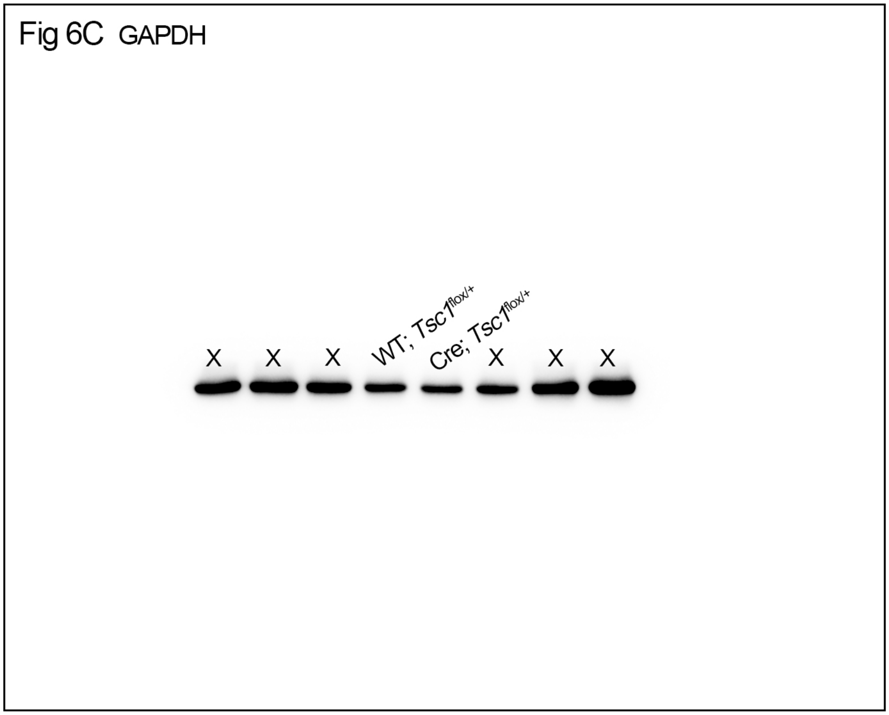
Fig 6C GAPDH
X
X
X
WT; Tsc1flox/+
Cre; Tsc1flox/+
X
X
X
